# The Experiences of Young People with Intellectual Disability, Parents and Professionals in Relationships and Sexuality Education Programmes: Findings from a Qualitative Study

**DOI:** 10.3390/healthcare12111105

**Published:** 2024-05-28

**Authors:** Michael Brown, Mark Linden, Lynne Marsh, Maria Truesdale, Fintan Sheerin, Freda McCormick

**Affiliations:** 1 School of Nursing and Midwifery, Queen’s University Belfast, 97 Lisburn Road, Belfast BT9 7BL, UK; m.linden@qub.ac.uk (M.L.); l.marsh@qub.ac.uk (L.M.); freda.mccormick@qub.ac.uk (F.M.); 2 Scottish Learning Disability Observatory, University of Glasgow, Glasgow G12 0XH, UK; maria.truesdale@glasgow.ac.uk; 3 School of Nursing and Midwifery, Trinity College Dublin, D02 PN40 Dublin, Ireland; fintan.sheerin@tcd.ie

**Keywords:** relationships, sexuality, education, intellectual disability, health, qualitative

## Abstract

People with intellectual disability want friendships and meaningful relationships, and some want intimacy. However, the expression of sexuality is an area where potential freedoms are often limited and restricted compared to their peers. While some relationships and sexuality education programmes do exist for this population, most focus on knowledge acquisition regarding sexuality and sex but lack in their focus on relationships, informed choices and decision-making. The aim of this study was to identify good practices and methods of delivery in relationships and sexuality education for children and young people with intellectual disability. A qualitative design was undertaken. Information about our study was distributed to eight special schools in the UK. Semi-structured interviews and focus groups were employed for data collection. Data from 37 pupils with intellectual disability, 11 parents and 16 healthcare and other professionals were thematically analysed. Following data analysis, three themes emerged: (i) seeking and sharing information; (ii) protecting and keeping safe; and (iii) learning for the future. The findings highlight that pupils are keen to learn about life changes and societal influences and want reliable information. Parents and professionals recognise that children and young people with intellectual disability will develop into adults and may be vulnerable when they leave the security of the school setting. They recognise that children and young people need to know about socialising, puberty, consent and contraception. Evidence-based programmes should be designed with these stakeholders to ensure children and young people with intellectual disability receive developmentally appropriate information to make happy and safe decisions about their relationships.

## 1. Introduction

The population of children and young people with intellectual disability is increasing and ageing, with more living into adulthood with a range of neurodevelopmental, physical, behavioural and mental health needs [1]. The changes are due to improvements in neonatal intensive care, wider health and social care services and access to care and support [2]. Children and young people with intellectual disability often have more complex support needs compared to typically developing children, particularly in understanding, learning and remembering new information and skills [3]. Some may require additional support with everyday activities such as communication, keeping safe and undertaking everyday tasks [4]. Consequently, many will require specialist services at some point in their lives [5].

There have been significant policy changes and developments over recent decades regarding the education of children and young people with intellectual disability, with moves towards inclusive education [6]. One recent educational development has focused on the right of children and young people with intellectual disability to experience relationships and express their sexuality [7]. While parents may be offered educational provision for their child to attend the same school as their typical peers [8], some children, due to their specific learning and support needs, may attend a blend of mainstream and special education provision [9]. For other children, notably those with the most complex educational and support needs, full-time attendance at a special education provision may be viewed as the most appropriate option [8]. A special school is a school that caters specifically to children and young people whose needs cannot be met with the provision and support provided by a mainstream school. This encompasses children and young people with many different types of educational needs [10]. 

The existing policy and research evidence recognises the rights of people with intellectual disability to lead fulfilling lives and make life choices [11,12,13]. People with intellectual disability want friendships and meaningful relationships, and some want intimacy [14]. However, the expression of sexuality is an area where potential freedoms are often limited and restricted compared to their typical peers [15]. People with intellectual disability are often misperceived as being either asexual, hypersexual or sexually immature [16]. Additionally, evidence highlights issues related to autonomy versus vulnerability, exploitation or risk of harm when supporting young people with intellectual disability to make decisions regarding their sexual activity [17]. There is a need to develop the understanding of families and professionals in education, social care and health services that many people with mild and moderate intellectual disability are interested in and actively engage in sexual relationships [14,18]. However, they possess less knowledge about relationships and sexuality, display more inappropriate sexual behaviours and often do not understand the consequences of engaging in unprotected sex [19]. Some practice unsafe sex, are less likely to use contraception, have an increased risk of having an unplanned pregnancy and have greater exposure to HIV and sexually transmitted infections (STIs) compared to typically developing young people [14,20]. Young people with intellectual disability may be at greater risk of sexual abuse and exploitation [21]. Some also experience difficulties in forming and maintaining relationships, resulting in loneliness and social isolation [20,22]. Current research evidence indicates that young people with intellectual disability may not have proper access to suitable relationships and sexuality education programmes [16]. While some relationships and sexuality education (RSE) programmes do exist for this population, most focus on knowledge acquisition regarding sexuality and sex, lacking in their focus on relationships, informed choices and decision-making [23]. Furthermore, existing programmes have not typically included the voices of children and young people with intellectual disability, parents and professionals involved in their education, care and support; therefore, potential RSE programmes should involve them. However, despite these issues, there remains a definitive gap in the delivery of RSE programmes that specifically address relationships and sexual needs and concerns for this population [14]. 

It is unclear what is currently being delivered in special schools and the process of evaluating and identifying outcomes in ways that meet the needs of children and young people with intellectual disability whilst also addressing parental concerns. Therefore, limits in consistency in provision and delivery may ultimately impact life choices and place the health and well-being of children and young people with intellectual disability at risk. Therefore, healthcare and other professionals have important health education roles in meeting the needs of children and young people with intellectual disability, including those related to relationships and sexuality [24]. They are well placed to work with children and young people with intellectual disability and their parents to ensure that RSE needs are identified and effectively addressed. 

The aim of this study was to explore the views and experiences of children and young people with intellectual disability, parents and professionals in the provision of RSE programmes in special schools across the UK. This paper presents a more detailed and comprehensive analysis of the study findings that were reported in part in the final report commissioned by the funder [25].

## 2. Materials and Methods

### 2.1. Design

A qualitative design involving semi-structured individual interviews and focus groups was adopted. All participants were provided with information about our study and a consent form that was completed prior to the interview or focus group. Accessible information and consent forms were provided to the pupils. The interviews and focus groups collected data on the views and experiences of participants in the delivery approaches regarding RSE for children and young people with intellectual disability in special school settings. 

### 2.2. Participants

Eight special schools across England, Northern Ireland (NI), Scotland and Wales were identified by the research team and, following an invitation, took part in this study. In each participating school, the principal, or a designated teacher, acted as a gatekeeper to identify pupils, parents and professionals who met the inclusion criteria and were willing to participate. The potential participants were then issued letters of invitation and information about our study. All participants were required to speak and understand English. Children and young people with profound and multiple intellectual disability were not included due to their communication abilities. The gatekeeper in each school made this determination based on their detailed knowledge of the pupils’ individual capabilities to participate in this study. Health professionals were approached through the existing contacts of the research team. 

Although not a requirement, all pupils and professionals had experience participating in relationships and sexuality education. 

Over a period of 13 months from February 2022 to February 2023, a total of 64 participants, comprising 37 pupils with intellectual disability aged between 12 and 19 years, 11 parents and 16 health, social care and education practitioners, provided informed consent and subsequently took part in an interview or focus group. A further 26 who did not participate included 10 pupils who became ill or whose parents did not provide consent and 5 parents and 11 professionals who were contacted on a number of occasions and did not respond. Table 1 shows the demographics and groups of those who participated across the United Kingdom. 

### 2.3. Data Collection

A Microsoft Teams online interview took place with 13 pupils and a face-to-face interview with 4; most were supported by a member of education staff. Pupil interviews lasted between 10 and 22 min. The remaining 20 pupils took part in two focus groups for boys or girls, with each group having education staff present for additional support. The boys’ group had a total of 12 participants, lasting 34 min. The girls’ group comprised eight participants and lasted 39 min.

Semi-structured individual interviews took place with 11 parents of children and young people with intellectual disability and 16 health, social care and education practitioners. Each interview lasted between 14 and 51 min for parents and between 26 and 66 min for professionals. Interviews took place via Microsoft Teams, telephone or in person at the school or parents’ home. 

Two focus groups comprising five and four professionals took place via Microsoft Teams and lasted 53 and 33 min, respectively. 

The interview and focus group questions centred on the participants’ views on and experiences of RSE programmes, for example, “What is your experience of RSE programmes for your child?”; the identification of topics for inclusion in an RSE programme, for example, “What would you like your parents and teachers to tell you about relationships and sexuality?”; and the identification of examples of good practice and preferred methods of delivery, for example, “What do you think are the best methods to teach young people with intellectual disability about RSE?”. 

The study research fellow (FM), who had no prior interaction with the participants, conducted all interviews and focus groups. Participants did not receive any gratification for taking part in this study. 

All interviews were recorded and transcribed verbatim and then anonymised by removing all identifiable information and assigning a numerical identifier. 

### 2.4. Data Analysis

The research team read each transcript separately to gain an understanding of the participants’ views and experiences. The coding of data into categories was performed by the research fellow and checked for consensus by the first author. The analysis of the data was facilitated by the data management programme NVivo 12 [26]. As part of the analysis process, themes and subthemes were systematically identified. Consequently, the proposed themes and subthemes were then discussed by the research team, with disagreements resolved and consensus reached. The approaches used in the qualitative data analysis and synthesis were rigorously followed to ensure trustworthiness, dependability and credibility throughout the process [27]. 

## 3. Results

Three broad themes emerged following data analysis that were associated with the development, delivery and evaluation of RSE programmes. These comprised the following: (i) seeking and sharing information; (ii) protecting and keeping safe; and (iii) learning for the future.

### 3.1. Seeking and Sharing Information

The demand for information on RSE amongst the pupils was evident from the data. All pupils had participated in RSE programmes of various durations. In the absence of a defined curriculum, the topics addressed had extended from ‘how to be a good friend’ to more in-depth information about consent, sex and contraception. In addition to classroom teaching, some pupils had also received information from friends and family, either through informal conversations or proactive approaches. Others had searched the internet and watched YouTube videos for information they were curious about. 

There was an eclectic mix of professionals delivering RSE programmes to young people. Primarily, this involved teachers and teaching assistants from within their special school who had varying levels of knowledge, expertise and confidence in the subject. To assist with the delivery in special schools, external educators with expertise in RSE programme delivery were occasionally engaged. These ranged from school nurses and social workers from local health and social services to trainers from independent agencies specialising in RSE. 

Parent participants were supportive of special schools in the delivery of RSE programmes. All participants considered it important, though, to be kept informed of RSE programme content, what was being taught and when. In contrast, others had attended workshops within the special school to gain a detailed insight into the programme content and delivery. Some parents expressed a need to be prepared to support their children and young people at home in case they asked questions and sought more information, thereby highlighting their development needs.

*“I get my information from online, [teacher], or anyone else willing to teach me… I learn from other people.”*

(Pupil 2, age 15)

*“I learned about like proper consent from You Tube videos. And the school actually did, like, do like a presentation thing about it, which is nice.”*

(Pupil 13, age 17)

*“It’s important to learn because if you don’t know then you don’t know what’s happening to your body.”*

(Pupil 23)

*“This stuff’s so important. My kids think, mum, you are out of your mind, but like, why do you have to be so open. I am like, because no one was ever open with me and I want you guys to feel that it is not taboo. I want them to be able to, you know, they can giggle, but they need to know what’s appropriate to say and do.”*

(Parent 9)

*“It’s still almost seen as a taboo thing. I don’t know, maybe, yeah, certainly from the people I’ve worked with that have come out of schools, nobody has come forward talking about that subject, and I think just making it a little bit more relaxed would be helpful.”*

(Professional 16)

### 3.2. Protecting and Keeping Safe 

Parents were aware that their children and young people were potentially vulnerable to predators online and in the community, recognising the need to protect them and keep them safe. Some parents reported having involved the police and school after their child had experienced unpleasant interactions online. All parents and professionals agreed that online safety and awareness and safety in relationships should be included in RSE programmes. One parent expressed concerns about their child’s safety and what would happen to them when they could no longer provide care. 

The need to maintain a healthy and safe lifestyle was also recognised as important. Professionals and some parents were open to including information on consent, contraception and sexually transmitted infections (STIs) for young people. Some schools included information on health checks and the importance of breast and testicular screening, with one arranging ‘bra fitting’ trips to a local shop to emphasise the importance of looking after yourself. 

The children and young people engaged in RSE learning and most understood their responsibilities regarding the content delivered within the programmes as well as the possible implications and consequences on their lives in the future, such as unintended pregnancy. This was evident across all ages, particularly in respect of online safety, and, for some young people, the prospect of cyber bullying, ending up in a coercive and abusive relationship or having an unintended pregnancy were to be avoided.

*“It’s about keeping you safe. It’s about being aware of the world out there because my mum said there’s some bad people that might make you feel uncomfortable.”*

(Pupil 25)

*“I reckon it’s so important because like, it can be too late and then a baby comes. Then practically your childhood’s ruined because you have a baby and all your friends are going out to clubs or whatever, and you’re sitting in the house minding a baby.”*

(Pupil 33, age 15)

*“If he [son] can’t interact safely with people once I’m gone, then, you know, what happens.”*

(Parent 1)

*“Relationships, sexual education is important in one sense to help provide an understanding of one’s body, but also probably to provide a level of protection as well. Because if they don’t understand what’s appropriate or inappropriate, they don’t understand where the boundaries are, then ultimately, they can become left in very vulnerable positions and people don’t understand.”*

(Parent 5)

*“Our children should be allowed to access the community and have a full life and they need to be given the tools to protect themselves and know about what is right and wrong and what’s acceptable to them.”*

(Professional 8)

### 3.3. Learning for the Future

Both parents and professionals were aware that, as children and young people with intellectual disability matured and aged, the need for RSE took on greater importance. Discussions regarding puberty and the ageing process were openly conducted with pupils by all the professionals and some parents who were involved in the delivery of all the RSE programmes in school and at home. Some parents were proactive in educating their children, notably when a school had not focused on a specific issue and where they considered the information was important and required. This was particularly relevant for parents who had daughters with intellectual disability, where they had been proactive and creative in sharing information about the menstrual cycle and pregnancy prior to menstruation commencing. Awareness of different sexualities was discussed in most of the RSE programmes, with some pupils sharing their own experiences and sexuality. 

There was consensus that learning should continue into adulthood and throughout life. This was viewed as necessary to reinforce and build upon the education provided and develop more relevant knowledge as young people aged, formed new relationships and experienced different situations. The apparent absence of age-appropriate and evidence-informed RSE programmes for adults and older adults with intellectual disability was viewed as a gap that needed to be addressed.

*“I think sometimes there is that preconceived idea that people who have got learning disabilities are not going to have their own relationships. And we know that just isn’t true, but their relationships may be very different. And I think again, it’s looking at those wider parameters of what a relationship looks like. So, it’s not necessarily about sex, but it’s about all of the other components and that they’re all as equally important.”*

(Parent 11)

*“These children are going to turn into adults and you want them to be able to go out into the community and understand, you know, the socially appropriate behaviours and the way we live and understand their own feelings and their own sexualities. You know, if it wasn’t talked about, what if you had a child with learning disabilities who themselves was say transgender.”*

(Parent 2)

*“When my daughter leaves school, she will be going on to different day care facilities, or whatever, day programmes, that is the word I am looking, and she will be meeting new people. She will have new facilitators, new carers, so she has to learn. And she is out in the big bad world as well, so that learning has to continue.”*

(Parent 4)

*“I’m bi so I like girls and boys. Yeah, so, it like helps me know what I like and what I am attracted to.”*

(Pupil 28)

*“It’s not a school that makes a child, it’s not an individual that makes a child, it’s community that makes a child. And I think as well as getting great resources for school, we need to be doing better support for our parents in that as well and making it a fully holistic approach to it, because they need to be seeing it not just in schools but in their homes. In the next part of their life they go to, they need to see all those aspects and be able to explore it safely in those as well.”* (Professional 4)

## 4. Discussion

There have been significant policy changes and developments over the past fifty years regarding the rights of children with disabilities, including the right to inclusive education and additional support [11,28]. These developments have focused on both inclusion in mainstream schooling and special school options [6,9]. For some parents, special schooling may be the preferred option for their child, particularly for those with more complex learning and support needs [8]. Whatever the model provided, the primary focus is on meeting the learning needs of the individual child. With the move towards inclusive, needs-led education for children and young people with intellectual disability, the role of RSE has been identified as an area requiring attention and development [7]. Curriculum developments are necessary to ensure the distinct needs and learning styles of children and young people with intellectual disability are recognised and addressed [23]. 

The findings from this study highlight the need for and importance of children and young people with intellectual disability participating in RSE programmes that are both accessible and specific to their needs and concerns. The study findings have sought to bring together and report on the perspectives of children and young people with intellectual disability, their parents and the professionals involved in their education, care and support. All have unique and important perspectives relevant to the development and content of RSE programmes and their planned delivery. From the study findings, it is evident that all participants were of the view that RSE programmes play an important role in the development of knowledge and understanding regarding relationships and the expression of sexuality. Many children and young people with intellectual disability have developed information technology (IT) skills, seek information and already engage in the use of social media. Providing support and access to RSE that enables informed decision-making and choices is a fundamental right of children and young people that needs to continue across their lifespan. Parents recognise the need for RSE programmes and the important role they play in educating children and young people with intellectual disability and in keeping them safe. Professionals appreciate the value and benefits gained by children and young people with intellectual disability from RSE programmes, recognising the requirement to both standardise and tailor content based on their needs and level of intellectual impairment. Collectively, the findings from the current study and wider research evidence have implications for future policy, practice, education and research.

### 4.1. Implications for Policy

In the past few years, with the strategic policy focus on RSE programmes being integrated within the school curriculum, there has been an opportunity to ensure that all developments and initiatives are fully inclusive of and reflect the needs and concerns of children and young people with intellectual disability, their parents and the professionals involved in the design, delivery and evaluation [29]. To reflect local policy, government and education providers should ensure there is specific reference to the distinct education and support needs of children and young people with intellectual disability and how they will be addressed and integrated within the curriculum. To effectively support RSE programme development and implementation in special school settings, agreement regarding core content that proactively includes the voices of children and young people with intellectual disability and their parents is necessary to ensure that all issues and concerns are recognised and responded to [30]. This is important from the outset, as particular aspects of RSE programme content may be viewed as contentious by some, such as capacity and consent, same-sex relationships and social media concerns such as pornography, sexting and cyber bullying [31,32]. Two specific policy areas require further attention and development. The first is the evaluation of the impact and outcomes achieved over time by participating in an RSE programme [33,34]. While children and young people with intellectual disability report enjoyment and enthusiasm from participating in RSE programmes, it remains to be established the extent to which learning can be generalised to real-life situations [34]. Identifying the impact of participation on concerns such as STIs, unintended pregnancy and child protection issues is essential. These are areas to focus on in future research to identify the impact on forming and sustaining relationships, health and well-being and longer-term outcomes [35]. The second relates to the importance of recognising and responding to the needs of adults and older adults with intellectual disability. To date, there has been limited attention given to RSE programmes that are reflective of the needs and aspirations of this cohort, and this is an area requiring policy attention and development [36].

### 4.2. Implications for Practice

From a practice perspective, there are implications arising from the study findings. Practitioners need to be prepared with the necessary knowledge and skills to effectively identify content relevant and appropriate to children and young people with intellectual disability in the development and delivery of RSE programmes [9,37]. To enable effective delivery, practitioners need time to engage with and involve children and young people with intellectual disability and their parents in the design and delivery of the programme [24]. With the diversity of populations and communities, practitioners involved in delivery need to identify and address cultural and religious beliefs in a way that is sensitive and acceptable [38]. Careful planning and involvement from the outset can help to answer questions, allay concerns and maximise benefits [35,38]. From a practice development perspective, access to networks of professionals engaged in RSE programmes can enable the sharing of best practices and help to build knowledge and confidence [33]. Current evidence highlights that the programmes are enjoyable for participants, yet it is less clear how learning is applied and generalised to the realities of life [37]. An important finding from this and other studies is the need to build in an evaluation of the learning gained by children and young people with intellectual disability from participating in RSE programmes from the outset. Therefore, practitioners need to consider and identify, as an integral part of planning, how the learning from participation will be evaluated [29]. Consideration also needs to be given to obtaining the views and experiences of parents regarding the benefits derived from participation and areas where further learning may be required [37].

### 4.3. Implications for Education

With the focus on integrating RSE programmes within the school curriculum as a core subject, there is a need to identify and meet the education and development needs of children and young people with intellectual disability, their parents and practitioners. Children and young people with intellectual disability require access to RSE programmes that are responsive and reflective of their needs, hopes and aspirations [39]. The education needs and concerns of parents and families also need to be identified and included [14,18]. RSE programme development, delivery and evaluation should be central to the education of practitioners. This is necessary and important as the findings from this study and the wider research literature highlight that practitioners often lack knowledge, skills and confidence around RSE [36,39]. Therefore, it is important to provide training at an undergraduate preparation level for teachers, social workers, nurses and others, integrating core content across the curriculum. This can start with the concepts of friendships and relationships, moving on to consider sexuality as young people with intellectual disability grow, mature and move into adulthood. At a postgraduate level, there is the opportunity to further develop this education [36].

### 4.4. Implications for Future Research

Conventions and statements regarding the rights of all people with disabilities have begun to positively impact the lives of some people with disabilities, including children, adults and older people with intellectual disability [11,28]. Inclusive to them are their relationship and sexuality needs and their right to have them identified and effectively addressed. The evidence base of what works to produce positive individualised outcomes must expand as a result of this research [40]. The research evidence, theories and behaviour change models used in the development of RSE programmes are an under-researched area and one that requires considerable attention. Undertaking research studies involving statistically appropriate samples to evidence significant effects is challenging [14,41]. To address these challenges, future RSE studies should, for example, adopt cluster randomised designs involving groups of special schools for children and young people with intellectual disability. Using this approach would allow for the effect of an RSE programme intervention on an entire school and a larger cluster to be identified. Children and young people and their parents want to be involved in and at the heart of RSE programme development, another area ripe for research focus. An important gap in the existing research evidence relates to the wider long-term benefits and outcomes derived from participating in an RSE programme. Future studies could identify, for example, reductions in unintended pregnancies, decreases in sexually transmitted infections and safeguarding concerns. Undertaking research in these areas will increase the evidence of outcomes achieved, the impact that RSE has on the lives of children and young people with intellectual disability in supporting informed decisions and how this can improve their quality of life.

### 4.5. Strengths and Limitations

The current study promotes the voice of children and young people with intellectual disability, parents and professionals regarding their experiences with RSE and its future requirements. The findings from this study add to our understanding of and provide further insights into the acceptance, content and delivery approaches of RSE for children and young people with intellectual disability, specifically in special school settings. A strength of this study is the participation of children and young people with intellectual disability. This is important as their views and experiences have been relatively silent in the research literature so far. The range of professionals who participated in this study reflects those with direct knowledge and experience of delivering RSE programmes to children and young people with intellectual disability. Some of the interviews with children and young people with intellectual disability were shorter than those with adults, which was primarily due to communication issues and their level of ability to engage in the interview more fully. However, including their views and experiences is vital in an area that has attracted limited attention thus far and is a particular strength of this study. Although the response rate from parents was good, it is recognised that there may be bias in the sample. Parents who participated in this study, for example, may not be fully reflective of the wider parent population and their views. While significant attempts were made to recruit all participants from across sites, it proved challenging, notably for some professionals and parents.

## 5. Conclusions

The evidence base regarding relationships and the expression of sexuality in children and young people with intellectual disability is growing and evolving. These developments are important and necessary by way of ensuring equality and protecting the rights of this, at times, vulnerable population. Failure to ensure that all children and young people with intellectual disability have access to RSE places them at potential risk of harm and avoidable sequelae that may have significant implications for their health and well-being, not just in childhood and adolescence but across their lifespan and into adulthood. What is apparent from the findings of the current study is that children and young people want and need access to education and support to enable the development of friendships and relationships and express their sexuality. Likewise, parents of children and young people want their children to participate in RSE programmes, recognising that RSE is an empowering process and experience that provides information to enable choice and informed decision-making. Professionals involved in the education, care and support of children and young people are key to the effective delivery and evaluation of RSE programmes specific to the needs of the population in special school settings. There is a need for further work to be undertaken to develop and empirically test RSE programmes that are sensitive and specific to the needs of these children and young people.

## Figures and Tables

**Table 1:** Demographics and groups of participants across the United Kingdom.

Region	Pupils	Parents	Professionals	Total
England	5	3	5	13
Northern Ireland	24	6	8	38
Scotland	5	0	1	6
Wales	3	2	2	7
Total	37	11	16	64

## Data Availability

Data are contained within the article.
